# Incidence and Risk Factors of Osteonecrosis of Femoral Head in Multiple Myeloma Patients Undergoing Dexamethasone-Based Regimens

**DOI:** 10.1155/2020/7126982

**Published:** 2020-05-16

**Authors:** Xinjie Wu, Chuanying Geng, Wei Sun, Mingsheng Tan

**Affiliations:** ^1^Peking University China-Japan Friendship School of Clinical Medicine, Beijing 100029, China; ^2^Department of Orthopedic Surgery, China-Japan Friendship Hospital, Beijing 100029, China; ^3^Multiple Myeloma Research Center of Beijing, Beijing Chao-Yang Hospital, Capital Medical University, Beijing 100020, China

## Abstract

**Objectives:**

To investigate the incidence and risk factors for osteonecrosis of femoral head (ONFH) in multiple myeloma (MM) patients undergoing dexamethasone-based regimens (DBRs).

**Methods:**

A retrospective study was conducted in MM patients administered DBRs between December 2012 and April 2015. Demographic, clinical, and laboratory data were extracted to compare between two groups. Incidence of ONFH were calculated and risk factors identified by both univariate and multivariate analysis.

**Results:**

The study group comprised 105 patients undergoing DBRs. Seven patients with ONFH after DBRs were classified as the ONFH group, and the other 98 patients without ONFH were included in the non-ONFH group. Incidence of ONFH was 6.7%. Median age of developing ONFH was 51 years (45–64), and the male to female ratio was 6 : 1. A total of 12 femoral heads were involved, including unilateral in 2 patients and bilateral in 5 patients. After the multivariate analysis, four risk factors were confirmed including male, younger age, cumulative dose of dexamethasone, and hyperlipidemia.

**Conclusion:**

The overall incidence of ONFH in MM patients treated with DBRs is 6.7%, and 4 risk factors are confirmed including male, younger age, cumulative dose of dexamethasone, and hyperlipidemia in our study.

## 1. Introduction

Multiple myeloma is a cancer originated from bone marrow plasma cells and influences more than 120,000 individuals each year worldwide [1, 2]. Dexamethasone-based regimens (DBRs) are the backbone of treatment in both first-line relapsed and refractory MM patients. Although DBRs have improved the prognosis of such disease significantly, it may also bring about some complications. Among them, osteonecrosis of femoral head (ONFH) is a rare adverse event in patients undergoing systemic corticosteroid treatment but one with considerable possibility of disability [3]. While corticosteroid-induced ONFH has been reported extensively in systemic lupus erythematosus, renal transplantation, and leukemia patients [4–6], there are few studies to identify the incidence and risk factors for ONFH in MM patients.

## 2. Materials and Methods

### 2.1. Patients

With institutional review board approval, we performed a retrospective study to investigate the incidence and risk factors for ONFH following DBRs for MM patients between December 2012 and April 2015. The inclusion criteria were in accordance with the diagnostic criteria of the International Myeloma Working Group [7]: at least 18 years of age and newly diagnosed MM with start of DBRs; no history of glucocorticoid use >2 g of prednisolone or its equivalent within a 3-month period; no ONFH sign in MRI before chemotherapy [8]; complete radiological data; and a follow-up period of more than 2 years. The exclusion criteria were the following: another tumor diagnosis; pediatric cases; history of radiation therapy for femoral head; patients with high risk of alcohol-associated osteonecrosis according to ARCO criteria [9]; poor compliance; unavailability of regular follow-up information; and insufficiency of the follow-up materials. Due to the fact that patients' information was anonymized and deidentified before analysis in this study, informed consent was not required.

### 2.2. Evaluation Indicators

Demographic and clinical data included gender, age of disease onset, weight, time to develop ONFH, symptom of ONFH, myeloma type, Durie-Salmon staging, International Staging System (ISS staging), hyperlipidemia, statin using, smoking, thalidomide using, bortezomib using, total days of dexamethasone treatment, cumulative dose of dexamethasone, and cumulative dose of dexamethasone per weight. Laboratory data included level of hemoglobin, albumin, *β*2-microglobulin, creatinine, calcium, lactate dehydrogenase, uric acid, and cytogenetics. All data were evaluated at diagnosis.

### 2.3. Statistical Analysis

Data analysis was carried out in the Stata software (version 15.0). The Shapiro-Wilk test was used to assess the normal distribution of data. Accordingly, data are presented as mean ± standard deviation (SD) or as median (range). Comparisons between groups were made using Student's *t*-test or Wilcoxon's rank-sum test, as appropriate. Categorical data were compared using the chi-squared test or Fisher's exact test. Variables with a *P* value less than 0.2 were evaluated in a multivariate logistic regression model. *P* values less than 0.05 were considered significant.

## 3. Results

Based on inclusion and exclusion criteria, a total of 105 patients were included in the present study. The baseline characteristics for non-ONFH and ONFH patients are shown in Tables 1 and 2. Seven patients (6.7%) developed ONFH after DBR treatment. In cases with ONFH, the median time to develop ONFH was 11.1 months (range, 1.6 to 76.4 months) and the male to female ratio was 6 : 1. A total of 12 femoral heads were involved, including unilateral in 2 patients and bilateral in 5 patients. The most common symptom of ONFH included pain on asymptomatic (*n* = 4, 57.1%), weight bearing (*n* = 3, 42.9%), pain at rest (*n* = 1, 14.3%), and limping (*n* = 1, 14.3%).

**Figure 1:**
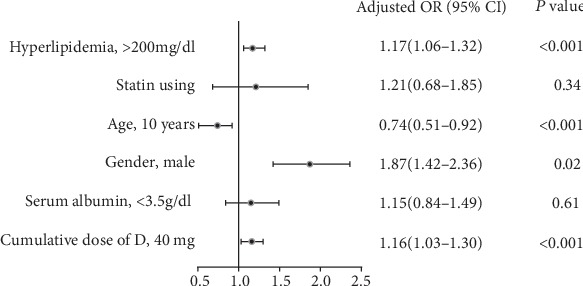
Risk factors for ONFH by multivariate logistic regression analysis. Note: total days of dexamethasone treatment and dexamethasone dose per weight were not included in the final multivariate logistic regression model due to its strong collinearity with cumulative dose of dexamethasone. ONFH: osteonecrosis of femoral head.

Six risk factors for ONFH were identified by univariate analysis, including age, gender, total days of dexamethasone treatment, cumulative dose of dexamethasone, dexamethasone dose per weight, and hyperlipidemia. Adjusted odds ratio (OR) for ONFH events from the multivariate logistic regression modeling is presented in Figure 1. Finally, 4 risk factors were confirmed including male, younger age, cumulative dose of dexamethasone, and hyperlipidemia. Notably, total days of dexamethasone treatment and dexamethasone dose per weight were not included in the final multivariate logistic regression model due to its strong collinearity with cumulative dose of dexamethasone.

## 4. Discussion

In present retrospective study, we found that ONFH occurred in 6.7% of patients with MM. Due to the retrospective nature of our study, the incidence is a little lower than the previous study (8.9%) [10]. Unlike the lower rates of osteonecrosis in renal allograft recipients (3.4%) [6], our results are comparable to the prevalence of osteonecrosis in other diseases treated with corticosteroids, such as SLE [4, 11]. Higher dose of corticosteroid in MM patients and immunosuppressive agents using such as cyclosporin or tacrolimus in renal transplantation may contribute to the result.

Five of 7 patients in our study got bilateral osteonecrosis in the femoral heads. 57.1% of the ONFH cases were found to have asymptomatic osteonecrosis, while 42.9% patients developed symptomatic osteonecrosis. Previous studies showed that 10-33% patients had symptoms of affected joints and monthly bisphosphonate prophylaxis may contribute to the low rate of symptoms [10, 12]. Notably, bisphosphonate, as a protective factor for hip, may increase risks for developing osteonecrosis of the jaw according to recent studies [13–15]. In addition, other drugs such as thalidomide and bortezomib were not found to be significantly related to the occurrence of osteonecrosis. Previous studies also showed that thalidomide was not a risk factor for ONFH despite its antiangiogenic properties [10, 16]. Bortezomib, as a proteasome inhibitor, was even reported that its anti-inflammatory effects may lead to the improvement of the jaw osteonecrosis in a MM patient [17].

**Figure 2:**
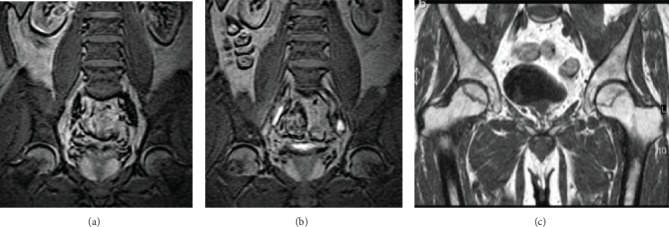
A 64-year-old male developed ONFH after DBRs treatment for 1.6 months. (a, b) Coronal magnetic resonance image before DBRs. (c) Coronal magnetic resonance image after cumulative dose of 200 mg dexamethasone for 1.6 months. ONFH: osteonecrosis of femoral head; DBRs: dexamethasone-based regimens.

**Figure 3:**
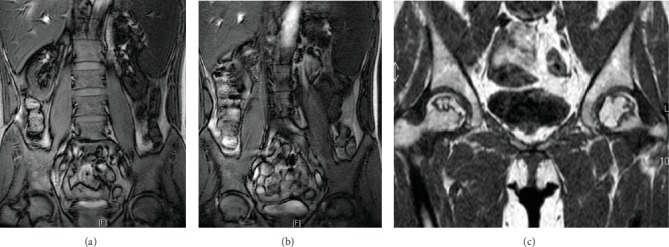
A 56-year-old male developed ONFH after DBRs treatment for 76.4 months. (a, b) Coronal magnetic resonance image before DBRs. (c) Coronal magnetic resonance image after cumulative dose of 4000 mg dexamethasone for 76.4 months. ONFH: osteonecrosis of femoral head; DBRs: dexamethasone-based regimens.

The median time to develop ONFH was 11.1 months. ONFH usually occurs within 2 years after the initiation of corticosteroid-based therapy. It may develop just for a short period of steroid apply. In our study, the shortest period to develop ONFH is 1.6 months (Figure 2). Unfortunately, ONFH can also develop after many years of corticosteroid therapy, and the longest period to occur is 76.4 months (Figure 3). Hence, asymptomatic changes of ONFH may initiate at any stage during the course of corticosteroid therapy, and early and periodic MRI examination could benefit patients.

For demographic characteristics, younger age and male are found to be related to ONFH, which is in accordance with previous studies [10, 18]. In human cells, there are around 6500 cytoplasmic binding sites per cell for dexamethasone and saturated around 50 nM [19]. However, receptors may decrease with age and result in insensitivity to corticosteroid. In general population, the male to female ration in osteonecrosis patients is 7 : 3 and even 8 : 1 [10, 20]. We also confirm being male as a risk factor for ONFH. Current studies demonstrate controversy regarding the relationship between cigarette smoking and ONFH. In our study, we did not find a significant difference in the risk of ONFH between two groups. A previous meta-analysis also showed that no significant difference in risk identified in light smokers (<20 pack-years) when compared with nonsmokers. This may contribute to the result of our study [21].

The mechanism of corticosteroid-induced ONFH remains obscure. According to literatures, dexamethasone is 6-fold toxic to bones compared with prednisolone, particularly when applied continuously [5, 22]. Although higher dose of dexamethasone may raise anti-MM response, it may also induce ONFH. We found that OR for ONFH is 1.16 when cumulative dose of dexamethasone increases 40 mg. Thus, judicious and minimizing use of dexamethasone and detection at early stage may help decrease the incidence of ONFH. Notably, for some cases, even small doses of corticosteroid could cause osteonecrosis and genetic predisposition may contribute the result. Genetic polymorphisms in many genes including SERPINE1, VEGF, VDR, CYP3A4, ACP1, and NOS3 are reported to be associated with ONFH [23]. These genes also play an important role in MM susceptibility and drug effects. Basmaci et al. [24] reported that the TNF*α* gene polymorphism (-308) GG genotype and NOS3 (+894) TT genotype were more common in the MM group compared to healthy controls. NOS3 (VNTR) AA and NOS3 (+894) GG genotypes were decreased in the MM group in contrast. Chen et al. [25] found that the A allele of rs699947 within VEGF and T allele of rs2228570 within VDR gene were all associated with increased MM risk. Hence, polymorphisms in such genes may increase the rate of osteonecrosis in MM patients.

It remains controversial whether hyperlipidemia influences the development of steroid-induced osteonecrosis [26–29]. Based on our data, there is a significant association between hyperlipidemia and osteonecrosis. Kuroda et al. reported that high triglyceride level is an important risk factor for silent ONFH in patients with SLE [27]. In addition, Mogensen et al. found that both hypertriglyceridemia and hypercholesterolemia are related to the development of osteonecrosis in children and young adults with acute lymphoblastic leukemia [28]. However, Calvo-Alen et al. reported a protective effect for hyperlipidemia in development of symptomatic osteonecrosis in lupus patients [30]. In a recent study, Zhao et al. found that preexisting hypercholesterolemia does not increase the risk of developing osteonecrosis in rabbits and lanolin-rich diets may be a protective factor [29].

Previous studies have reported that statins may improve hyperlipidemia and enhances femoral head neovascularization through suppresses PPAR*γ* expression and activates Wnt3a/LRP5/b-catenin/RUNX2 signaling pathway in steroid-induced animal models [31–33]. In addition, PPAR*γ* receptor protein is also expressed in myeloma cells. Previous studies showed that its natural and synthetic ligands induce apoptosis of tumor cells [34, 35]. Hence, PPAR*γ* ligands may represent a novel therapy for MM. However, clinical studies reported different and controversial results for the effect of statin in osteonecrosis patients [36, 37]. In our study, we did not detect a significant association between statin and ONFH.

There are several limitations in present study. Firstly, due to the retrospective nature, it may influence the true incidence of ONFH in MM patients and identification of risk factors. Secondly, treatment protocols may evolve and dose of dexamethasone may vary over the course. In spite of these limitations, our results successfully provide relevant information on a rare complication.

## 5. Conclusion

In summary, the overall incidence of ONFH in MM patients treated with DBRs is 6.7%, and 4 risk factors are confirmed including male, younger age, cumulative dose of dexamethasone, and hyperlipidemia in our study.

## Figures and Tables

**Table 1:** Continuous variables of patient characteristics.

	Non-ONFH (*n* = 98)		ONFH (*n* = 7)		
Continuous variable	Median	Range	Median	Range	*P*
Age (years)	56	32-79	51	45-64	0.03
Weight (kg)	65.82	50-82	67	51-78	0.28
Time to develop ONFH (months)	—	—	11.1	1.6 to 76.4	
Total days of D treatment	40	8-116	56	20-200	0.002
Cumulative dose of D (mg)	800	160-1740	1120	400-4000	0.002
D dose/weight (mg/kg)	12.6	2.7-27.6	16.7	6.1-55.6	0.009
Hemoglobin (g/l)	71.1	54.8-111.7	73.8	53.8-85.3	0.29
Serum albumin (g/dl)	4.3	2.9-4.8	3.7	2.9-5.1	0.08
Serum *β*2-microglobulin (mg/dl)	4.4	2.1-47.6	7.5	3.1-36.5	0.34
Serum creatinine (*μ*mol/l)	116.14	48.74-175.18	88.71	76.33-157.89	0.66
Serum calcium (mmol/l)	3.38	1.89-4.23	3.49	3.12-5.56	0.52
Lactate dehydrogenase (IU/l)	263.5	40-1700	451	20-1179	0.38

ONFH: osteonecrosis of femoral head; D: dexamethasone.

**Table 2:** Categoric variables of patient characteristics.

	Non-ONFH (*n* = 98)	ONFH (*n* = 7)	
Categoric variable	Number	Number	*P*
Gender			
Male	46	6	0.04
Female	52	1	
Symptom of ONFH			
Asymptomatic		4	
Pain on weight bearing		3	
Pain at rest		1	
Limping		1	
Myeloma type			
IgG	27	1	0.68
Light chain	43	4	
IgA	21	1	
Nonsecretory	7	1	
Durie-Salmon staging			
I	7	0	0.67
II	7	1	
III	84	6	
A	90	7	0.43
B	8	0	
ISS staging			
I	21	0	0.27
II	39	2	
III	41	5	
Hyperlipidemia, >200 mg/dl			
Yes	33	5	0.04
No	65	2	
Uric acid, >420 *μ*mol/l			
Yes	32	4	0.19
No	66	3	
Statin			
Yes	21	3	0.19
No	77	4	
Thalidomide			
Yes	40	3	0.61
No	48	4	
Bortezomib			
Yes	81	6	0.84
No	17	1	
Cytogenetics			
Normal	35	2	0.70
Abnormal	63	5	
Smoking			
Yes	35	4	0.26
No	63	3	

ONFH: osteonecrosis of femoral head; ISS: International Staging System.

## Data Availability

The data used to support the findings of this study are available from the corresponding author upon request.
